# Investment in Seed Physical Defence Is Associated with Species' Light Requirement for Regeneration and Seed Persistence: Evidence from *Macaranga* Species in Borneo

**DOI:** 10.1371/journal.pone.0099691

**Published:** 2014-06-13

**Authors:** Pimonrat Tiansawat, Adam S. Davis, Mark A. Berhow, Paul-Camilo Zalamea, James W. Dalling

**Affiliations:** 1 Department of Plant Biology, University of Illinois, Urbana, Illinois, United States of America; 2 Global Change and Photosynthesis Research Unit, United Department of Agricultural – Agricultural Research Service, Urbana, Illinois, United States of America; 3 National Center of Agricultural Utilization Research, United Department of Agricultural – Agricultural Research Service, Peoria, Illinois, United States of America; 4 Smithsonian Tropical Research Institute, Ancon, Republic of Panama; Key Laboratory of Tropical Forest Ecology, Xishuangbanna Tropical Botanical Garden, Chinese Academy of Sciences, China

## Abstract

The seed stage is often critical in determining the regeneration success of plants. Seeds must survive an array of seed predators and pathogens and germinate under conditions favourable for seedling establishment. To maximise recruitment success plants protect seeds using a diverse set of chemical and physical defences. However, the relationship between these defence classes, and their association with other life history traits, is not well understood. Data on seed coat thickness and fracture resistance, and the abundance and diversity of potential defensive compounds were collected for 10 tree species of *Macaranga* from Borneo. The data were used to test whether there is a trade-off in physical versus chemical defence investment, and to determine how investment varies with seed mass, and light requirement for regeneration. Across species there was no correlation between seed coat thickness and abundance of potential defensive compounds, indicating the absence of a direct trade-off between defence classes. While chemical defences were not correlated to other traits, physical defences were positively correlated with light requirement for regeneration. For a subset of five *Macaranga* species we evaluated the relative investment in chemical and physical defence to seed persistence in the soil, measured as the time to half initial seed viability (seed half-life). Half-life was negatively related to the ratio of potential defensive compound abundance to seed coat thickness, suggesting that species with long persistence invested in physical defence more than stored chemical defences. These results indicate that investment in seed defences are associated with species' light requirements for regeneration, rather than scaling positively with seed mass. Furthermore, chemical defences, although highly variable among species, do not appear to be critical to long term persistence of *Macaranga* seeds, and may be important in defending seeds from natural enemies distinct from those found in the soil.

## Introduction

Natural enemies strongly influence plant population growth, plant traits, and the local and regional distribution of plant species [1], [2], [3]. Many of these effects are most strongly exerted at the seed stage [4], [5]. Since the seeds of most plant species need to contain sufficient nutrients to support seedling establishment, they represent rich resources that are consumed by a wide range of animals [6], [7], [8] while also providing a substrate for microbial infection in the soil [9], [10], [11]. As a consequence, maternal resources are allocated not only to provisioning seeds, but also to the protection of seeds from predators and pathogens [12], [13].

Seed defences are often sub-divided into traits that provide physical or chemical protection. These protective traits are usually associated with seed-enclosing structures (e.g., pericarp and testa; [14], [15]). Physical defences are provided by the thickness and fracture resistance of enclosing structures that protect against penetration by insect predators [16], or seed rupture when passed through the jaws and digestive tracts of dispersers [14]. However, while physical defences can provide effective mechanical protection against vertebrates and invertebrates) [17], [18] seed-protecting structures may still be consumed by predators and seeds may be susceptible to pathogen infection [19]. Therefore, to discourage predators and pathogens seeds may also develop other means of protection based on chemical defences.

Plants contain many classes of secondary compounds that potentially serve as chemical defences against predators and pathogens [20], [21], [22]. In seeds, several classes of compounds such as phenolics, and alkaloids have been reported to be to be effective in protecting against insects [23], molluscs [24], vertebrates [25], and fungal and bacterial pathogens [19], [20], [26]. In *Macaranga beccariana*, potential stored defensive compounds in a class of dihydroxyphenols such as gentisic acid, protocatechuic acid, and gamma-resorcylic acid have been found, especially in the seed coat (Tiansawat, unpublished data). The presence of potential defensive compounds in a hard seed coat suggests that *Macaranga* species invest their resources in both physical and chemical protection of their seeds.

Many theories have been developed to explain the distribution of defences across plant structures and among plant species on the basis that defences are costly to produce [27], [28], [29]. Consequently, the allocation of resources to defences is hypothesized to be subjected to trade-offs such that high investment in one defence type should reduce investment in others with similar effectiveness [30], [31], [32]. However, most studies that have explored trade-offs in plant defences have focused on the leaves of seedlings and adult plants (e.g., [30], [31], [32], [33], and it remains unclear whether a trade-off exists between physical and chemical defensive traits in seeds.

In addition to undergoing trade-offs, allocation to seed defences may also be habitat specific. In general, seedlings and adults of light-demanding species are characterized by a low investment in defensive traits relative to shade-tolerant species [2], [27], [34]. However, it is unclear whether defence allocation patterns post-germination also extend to seeds. Light-demanding species often form persistent soil seed banks that require seeds to survive long exposure to post-dispersal seed predators and pathogens [35], [36]. Therefore, prolonged seed persistence may instead be associated with higher investment in defence.

The relative investment in physical and chemical defences associated with prolonged seed persistence remains unclear. Previous work with 80 native British species and six neotropical pioneer species has shown that longer persistence in the soil is associated with increased investment in chemical protection [37], [38]. In contrast, the most persistent temperate weed species allocate relatively more resources to physical defence than those species with short persistent seed banks [39], [40]. These contrasting results suggest complex relationships may exist linking seed size, persistence and seed chemical and physical defences, and may also vary among ecosystems and phylogenetic clades.

In this study, three questions were asked regarding the distribution of seed defences among species: First, does a trade-off exist among species between the physical defences and the quantity of potential defensive compounds? Second, does seed mass variation underlie interspecific differences in seed physical and chemical defences? We hypothesized physical defences should become more effective as seed mass increases, because the absolute thickness of seed protecting structures will scale positively with seed mass even if investment in physical defence is unchanged. Furthermore, if defence investment is subject to trade-offs, then larger-seeded species will be less dependent on chemical defences than smaller-seeded ones.

Third, does light requirement for regeneration influence the distribution of seed physical and chemical defences among species? We hypothesized that light-demanding species that form persistent seed banks will have greater investment in both physical and chemical defences than shade-tolerant species that germinate soon after dispersal. Alternatively, if physical damage is a primary cause of seed loss, then the half-life of seeds in the soil and the ratio of chemical to physical defences will be negatively correlated, indicating species that persist longer in the soil will invest more in physical defence relative to chemical defence.

## Materials and Methods

### Study species and site

In ever-wet paleotropical forests, the genus *Macaranga* serves as a unique system for studying the relationships among seed defence, habitat requirements and seed persistence. For the 10 Macaranga species included in this study seed mass ranged from 1.72 to 64.09 mg, and in habitat preference from shade-tolerant shrubs to light-demanding trees. Mature fruits of 10 *Macaranga* species (Table 1) were collected between July 2009 – September 2011 from primary and secondary forests of Lambir Hills National Park (hereafter Lambir), Sarawak, Malaysia (NW Borneo) (4°12' N, 114°02' E) (Permit number NCCD.907.4.4(V)-97 and NCCD.907.4.4(Jld.Vl)-84). The mean temperature is 26°C. Lambir receives annual rainfall ranging between 2100 and 3300 mm [41] with all months averaging >100 mm [41]. Lambir has no clear seasonality; however, unpredictable short dry spells (<1 month) often occur throughout the year [42].

**Table 1:** Summary of seed shape and surface sculpturing, species crown illumination index (CI index), and physical and chemical defence determined for 10 *Macaranga* species.

Species	Seed shape and sculpturing*	CI**	Seed dry mass (mg)		Seed coat thickness (µm)		Total mass-standardized peak area (10^8^ mV×min/g sample)	Maternal source
*Macaranga*			Mean	SE†	Mean	SE‡		
*beccariana*	S	3.20	12.19	1.70	319.9	4.8	3.47	3
*hypoleuca*	S	3.20	19.58	.	375.8	5.6	6.69	1
*umbrosa* 1	O	2.00	60.47	.	126.0	1.2	0.61	1
*lamellata*	O	1.80	64.09	.	130.8	2.9	3.06	1
*bancaca* 2	O	3.05	18.73	0.61	218.3	4.7	19.60	3
*trachyphylla*	O	3.05	20.41	0.14	234.3	24.6	14.92	2
*hullettii*	O	2.05	24.72	1.57	178.0	3.1	13.72	3
*havilandii*	O	2.75	33.22	0.66	171.3	1.8	22.11	2
*gigantea*	L	3.50	17.60	1.81	410.0	3.3	3.45	3
*winkleri*	V	3.70	1.72	0.13	94.7	2.9	0.47	3

*Seed shape and surface sculpturing from Davies 2001 [63] and Whitmore 2008 [64].

**Figure 1:**
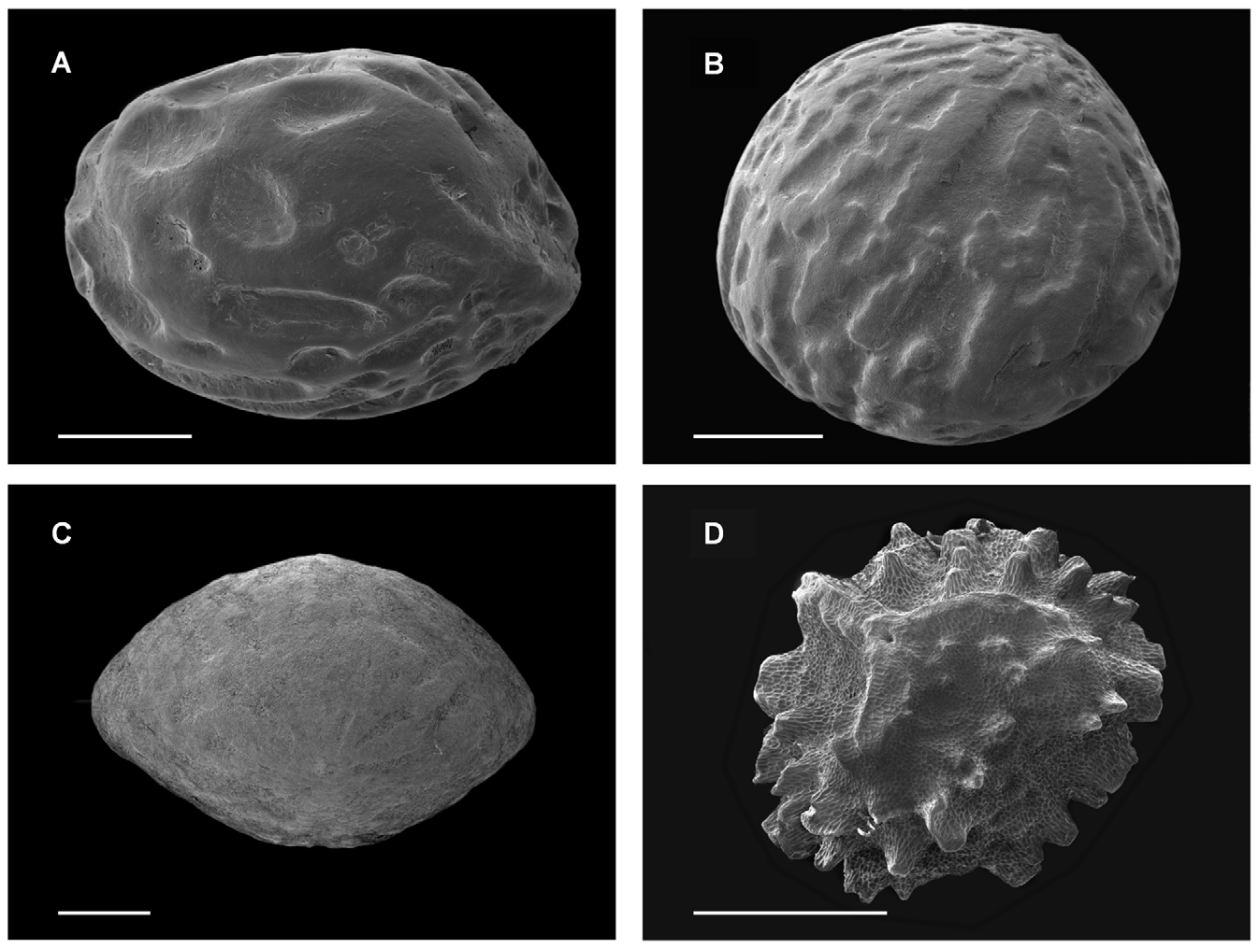
Four types of seed shape and sculpturing of Bornean *Macaranga* species (scale bar: 1 mm) – (a) *M. hypolueca*: spheroidal or slightly flattened with large shallow round pits (S), (b) *M. bancanca*: ovoid or subtriangular-ovoid with shallow grooves (O), (c) *M. gigantea*: lenticular with shallow grooves (L), and (d) *M. winkleri*: ovoid shape with coarsely verrucose surface (V).

O: ovoid or subtriangular-ovoid with shallow grooves, S: spheroidal or slightly flattened with large shallow round pits, L: lenticular with shallow grooves, V: ovoid shape with coarsely verrucose (peg-like structure) surface (Figure 1).

**Mean crown light index (CI) of the seedling and sapling stages (diameter size class of 0–4 cm) from Davies et al. (1998) [44]. The index, ranging from 1 to 5, scores the source (lateral and vertical light) and relative amount of light available for individual crowns [43].

† Standard error among seed sources. Missing value indicates one maternal tree contributing the seeds.

‡ Standard error among seeds of pooled from maternal sources or seeds of one maternal source.

1 Previously reported as *Macaranga kingii* in Davies et al. 1998 and Davies and Ashton 1999.

2 Previously reported as *Macaranga triloba* in Davies et al. 1998 and Davies and Ashton 1999.

### Light requirement for regeneration and seed collection

The light requirement of species was represented by the crown illumination index (CI index), [43]. CI index scores were obtained from published literature [44], and were based on a visual assessment of the crown exposure to light. Species were classified on a scale of 1–5 from very shade-tolerant to highly light-demanding. Since light requirements may change during ontogeny [45], only the CI index of *Macaranga* individuals <4 cm diameter were used, and were obtained by averaging the value of CI index across the first two columns of Table 3 in [44].

For seed collection, mature fruits of 10 *Macaranga* species (Table 1) were collected between July 2009 and September 2011 from primary and secondary forests of Lambir. All fruits were collected directly from the crowns of at least two maternal sources (except one parent for fruits of the rare species *M. lamellata*, *M. umbrosa* and *M. hypoleuca*). The numbers of seed sources and seeds used in the study were relatively small. This is a consequence of low fruit availability during non-mast years, and further constraints on seed production that are a consequence of resource limitation to relatively large-seeded trees that grow beneath a forest canopy.

*Macaranga* seeds are enclosed with oil-rich arils. The presence of aril is recognized as a reward for seed dispersal agents. The soft nutrient-rich tissues are likely to be decomposed rapidly and would not persist long enough to affect persistence in the soil. Therefore, in this study examinations of physical and chemical defences, and seed persistence in the soil excluded the aril. Seeds (Figure 1) were cleaned the same day of collection to remove arils and to exclude non-viable seeds that floated in water. To measure seed dry mass, two replicates of 10 seeds from each maternal source were randomly sampled and were oven-dried at 65°C for three days and reweighed. For each species, seeds from different maternal sources were pooled, stored in a refrigerator, and shipped by air courier in airtight plastic bags to the University of Illinois for physical and chemical defence analyses.

### Measurement of seed physical defence

Quantitative differences in seed physical protection among species were represented by a measurement of seed coat thickness. In *Macaranga*, seed coat thickness was strongly correlated with seed fracture resistance, i.e. the minimum force required to initiate seed rupture (Appendix S1, Figure S1). Therefore, the seed coat thickness served as a good estimate of seed physical strength. Measurements of seed coat thickness were made using a dissecting microscope (ZEISS SteREO Discovery V8, Carl Zeiss Microscopy, Germany). Ten seeds of each species, (except *M. lamellata* and *M. umbrosa* for which five seeds were measured) were randomly selected and cut in half. The half-seeds were photographed under the dissecting microscope and seed coat thickness measured at three random points using graphic tools of ZEN 2011 imaging software (Carl Zeiss Microscopy, Germany). The mean of seed coat thickness was obtained for each species.

### Measurement of seed chemical defence

Rather than targeting a specific class of compounds, seed chemical defence investment was characterized as the quantity of potential defensive organic compounds in methanolic extracts of seed coat and may include phenols, flavonoids and alkaloids. Seeds were pooled from different maternal sources and the seed coats and seed contents separated by gently breaking seeds open in a mortar and pestle. The seed coats were then ground to a fine homogenate using a Wiley Mini Mill (model 3383-L10 Thomas Scientific, USA). For each species, three replicates of 0.1 g of ground seed coat were extracted in methanol (Appendix S2). The triplicates of methanol supernatant were then analysed for compounds using high performance liquid chromatography (HPLC; Appendix S3).

In this study, potential defensive compounds were not identified but were instead distinguished according to their retention times. An absorbance peak (at 280 nm), shown in a chromatogram, was used as a relative abundance measure for a series of unknown peaks. To compare compound abundance across samples, the area of each peak in each sample was first standardized to the mass of the seed coat sample.
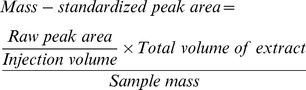
where peak area was measured in mV×min, injection volume was 25 µL, extract volume was 1.5 mL, and sample mass was measured in g. Mean peak areas were calculated from the three replicates per species. The mass-standardized peak area of all potential defensive compounds present in each species was then summed. In *Macaranga* the number of absorbance peaks and the mass-standardized peak area of potential defensive compounds were strongly and positively associated (Figure S2). Therefore, only the mass-standardized peak area of compounds was used as a measurement of the abundance of potential defensive compounds [46].

### Seed persistence in the soil

A burial experiment to determine the loss rate of seed viability over time was conducted at Lambir from July 2009 to November 2011. Due to the low availability seeds, this experiment was limited to five species: *M. bancana, M. beccariana*, *M. gigantea*, *M. trachyphylla* and *M. winkleri*. There were two maternal sources for *M. beccariana* and *M. winkleri*, and one maternal source each for *M. bancana*, *M. gigantea* and *M. trachyphylla*. For initial seed germination, at least two replicates of 30 seeds per maternal source were sown in Petri dishes lined with two layers of sterile cotton gauze pad (Dynarex Corporation, New York, USA) for eight weeks. The cotton gauze pad was saturated with 5 ml of tap water and the dishes were covered with lids. Dishes were checked daily, and water was added every 2–3 days to resaturate the gauze pad.

Buried seeds of each maternal source were placed in individual fibreglass netting (0.5 mm mesh size) bags together with 10 g of homogenized forest soil. Mesh bags retained the seeds but were permeable to fungi and small invertebrates [36]. Each bag of the same maternal source contained the same number of seeds; however, the number of seeds per bag varied among maternal sources depending on seed availability (15 to 30 seeds), and the number of seed bags per maternal sources ranged from 27 to 42 bags. The seed bags were buried 5 cm below the soil surface and 30 cm apart from one another in a common garden beneath the forest canopy. The burial site was in a shade environment (average R: FR of 0.14±0.04) of primary Dipterocarp forest canopy of Lambir at 102 m above the sea level. Daily air temperature ranged from 24.0 to 28.6°C (StowAway TidbiT temperature data loggers, Onset Computer Corporation, MA, USA).

Seed bags were recovered 1, 2, 3, 6, 12, 19, 22 and 28 months after burial. For some maternal sources with few seed bags, recovery periods of seed bags varied, but spanned the 28 months. Four to eight seed bags of each maternal source were retrieved at each time point. After recovery, the seed bag content, which was comprised of soil and seeds, was placed individually in a Petri dish lined with a piece of moist filter paper (No. 1 Whatman filter paper, GE Healthcare, New Jersey, USA). The Petri dishes were covered with lids and placed on the shelves in a growing house with 30% full sun (average R: FR 1.12). Averages of day and night time air temperature in the growing house were 31.0 and 23.5°C, respectively; the average diel temperature is 26.7°C. The seed bag contents were sprayed with water every three days to maintain moisture. Germination was scored over five weeks as visible radicle or hypocotyl emergence.

## Data Analysis

### Seed mass, light requirement for regeneration and seed defence

Unless stated, data were analysed using R version 2.15.3 [47]. To examine trait relationships across species, the pairwise Pearson correlation matrix was computed for seed dry mass, seed coat thickness, total mass-standardized peak area of potential defensive compounds, and crown illumination index (CI index). Phylogenetic analyses were also conducted on trait comparisons. The results accounting for phylogeny were qualitatively similar to the results of the cross-species analyses (Table S1 in Appendix S4).

### Ordination of potential defensive compound composition and light requirement

Principal coordinate analysis (PCO) (package *ecodist* version 1.2.7) [48] was used to position species in space according to differences in potential defensive compounds present in seed coats. The PCO is a metric multidimensional scaling method based on projection, which uses spectral decomposition to approximate a dissimilarity matrix of the distances between a set of points in a few dimensions [49]. The PCO is useful for biological data with many zeroes in the data set. Bray-Curtis index was used to calculate the dissimilarity matrix. The number of dimensions was fixed to two.

### Seed persistence in the soil

In our study, no dormancy was observed for any species when seeds were exposed to suitable environmental conditions. Therefore, seed germinability reflects seed viability. The relationship between seed germination percent (*y*) and time (*t*) was described as a negative exponential function [39], [50]. The exponential decay function was in the form of:
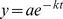
where *a* represents initial percent germination and *k* is the exponential decay constant. The time over which viability of seeds was reduced to half of initial viability, was used as a comparison index of seed persistence in the soil. Half-lives of seeds in the soil were estimated under the assumption of a constant rate of viability loss over time (*t*). Seed half-life in the soil (*t_0.5_*) for each species was calculated as *t_0.5_ = ln(0.5)/-k.*

To explore the relationship between seed persistence and seed defence, the ratio of total mass-standardized peak area of potential defensive compounds to seed coat thickness was used. This ratio provides a measure of the relative amount of investment to chemical and physical defence [39]. The log*_e_*-transformed ratio was regressed against log*_e_*-transformed seed half-life using a linear regression (*lm*).

## Results

### The trade-off between seed physical and chemical defence

**Figure 2:**
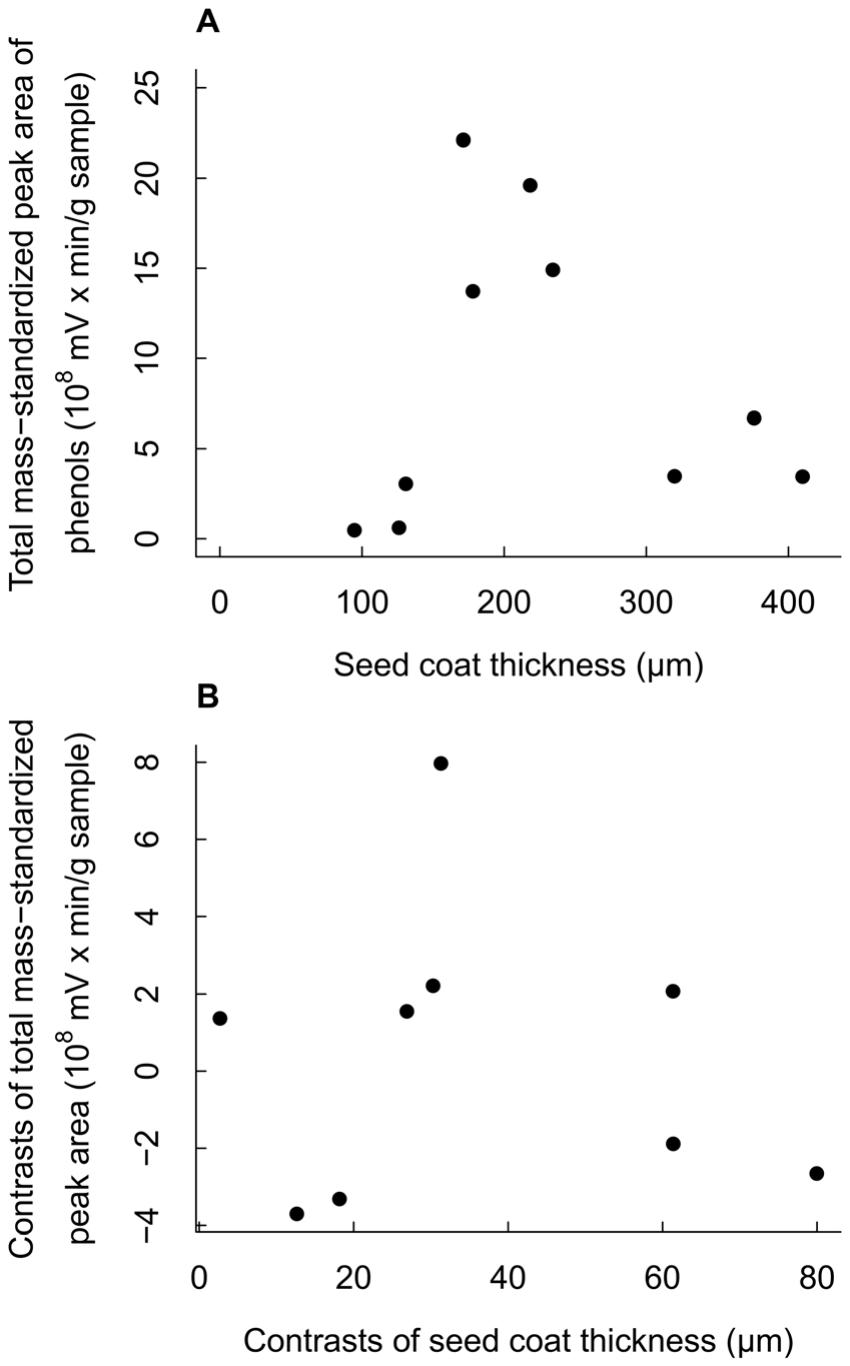
The cross-species analysis (a) and the analysis of phylogenetically independent contrasts (b) showed no linear relationship between seed coat thickness and total peak area of potential defensive compounds.

There was no significant correlation between seed physical and chemical defence either before or after controlling for phylogenetic relationships (Table 2, Figure 2). Species with both relatively thin (<150 µm) and thick (>300 µm) seed coats had relatively low quantities of potential defensive compounds compared to species with intermediate seed coat thickness (170–220 µm) (Figure 2A).

**Table 2:** Pearson product-moment correlation matrix between all paring seed attributes of 10 *Macaranga* species.

	Crown illumination index*	Seed dry mass*	Seed coat thickness*
Seed dry mass	**−0.87**		
Seed coat thickness	0.50	−0.41	
Total mass-standardized peak area	−0.04	−0.13	−0.04

* The coefficients with significance *P*<0.001 are indicated in boldface.

### Seed physical defence, light requirement for regeneration, and seed mass

**Figure 3:**
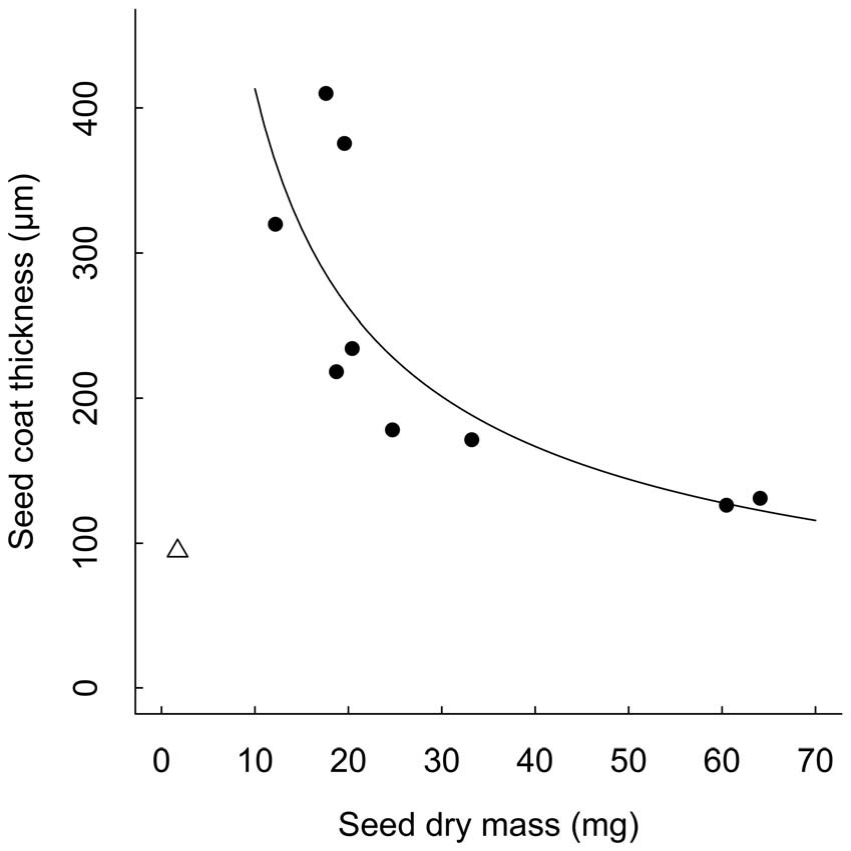
The relationship between seed mass and seed coat thickness for 10 *Macaranga* species. The outlier *M. winkleri* data (▵) was excluded from the model (log(*Y*) = 7.5328–0.6563×log(*X*), *n* = 9, *R^2^* = 0.7322, *F _(1,7)_* = 19.14, *P* = 0.003).

The light requirement for regeneration, represented by the CI index of seedling and sapling stages, ranged from 1.80 (shade-tolerant) to 3.70 (strongly light-demanding) across the 10 *Macaranga* species (Table 1). The CI index was strongly negatively correlated with seed mass (Table 2), indicating that more light-demanding species produced smaller seeds. There was also a significant negative relationship between seed coat thickness and seed mass, where *M. winkleri*, the only *Macaranga* species with a sculpted seed coat, was a significant outlier (Figure 3).

Because seed mass and CI index were strongly correlated (Table 2), their association with thickness of seed coat was examined using partial correlation. When controlling for CI index, seed coat thickness was uncorrelated with seed mass (Partial correlation coefficient  = −0.04, *n* = 9, *P* = 0.92). When controlling for seed mass, and excluding the outlier *M. winkleri*, seed coat thickness was positively associated with CI index (Partial correlation coefficient  = 0.66, *n* = 9, *P* = 0.03), indicating that shade-tolerant species had relatively thinner seed coats than light-demanding species.

### Seed chemical defence of Macaranga species

**Figure 4:**
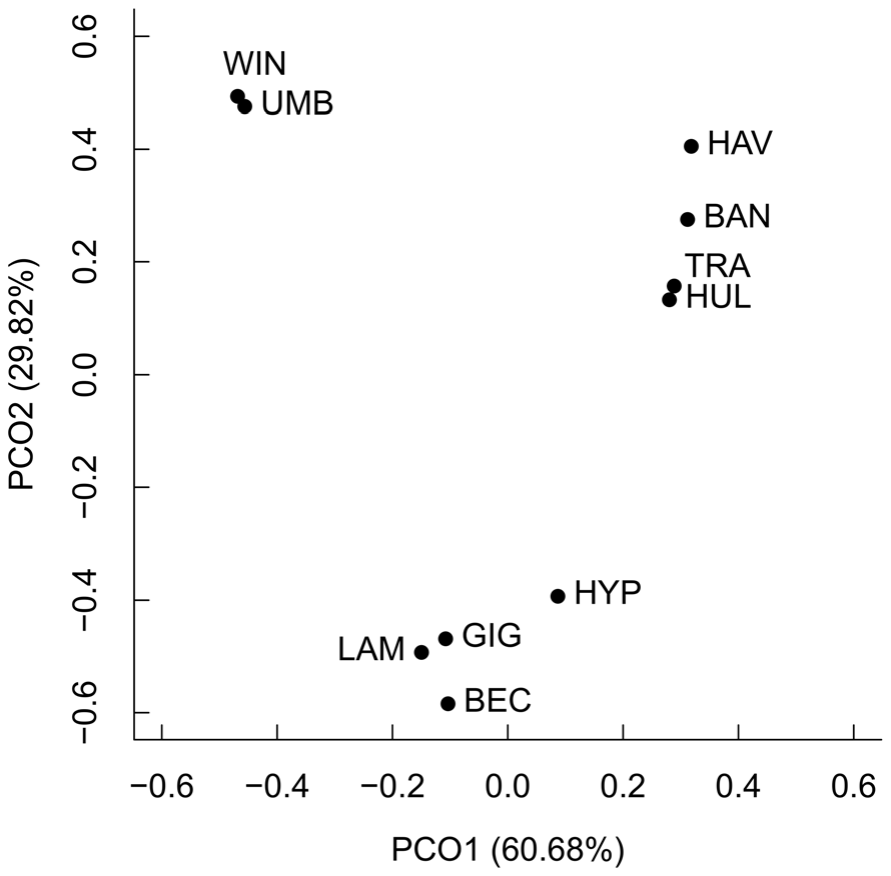
Principal coordinate analysis (PCO) plot showing similarity of potential defensive compound composition of 10 *Macaranga* species - *M. bannacana* (BAN), *M. becciariana* (BEC), *M. gigantea* (GIG), *M. havilandii* (HAV), *M. hullettii* (HUL), *M. hypoleuca* (HYP), *M. lamellata* (LAM), *M. trachyphylla* (TRA), *M. umbrosa* (UMB), and *M. winkleri* (WIN). The two axes explained 90.50% of the viability in the original dissimilarity matrix.

A total of 23 distinct peaks were recorded likely representing 23 compounds in the 10 *Macaranga* species. The lowest peak diversity was in *M. winkleri* with one compound peak, while *M. trachyphylla* and *M. havilandii* had the highest diversity with 20 distinguishable peaks. Total mass-standardized peak area of potential defensive compounds was not associated with CI index or seed mass (Table 2). The principal coordinate analysis (PCO) used to position species according to similarity in potential defensive composition revealed three distinct species groups (Figure 4): (1) *M. beccariana*, *M. gigantea*, *M. hypoleuca* and *M. lamellata*, (2) *M. bancana*, *M. trachyphylla*, *M. havilandii* and *M. hullettii*, and (3) *M. winkleri* and *M. umbrosa*. These groups were not associated with seed mass, or light requirement for regeneration.

### Seed persistence, seed mass, light requirement for regeneration and defence

Data on persistence (half-life) (Figure S3) of seeds in the soil were available for five *Macaranga* species: *M. bancana* (0.36 year), *M. trachyphylla* (0.48 year), *M. beccariana* (0.77 year), *M. winkleri* (1.26 years), and *M. gigantea* (1.78 years). When controlling for seed mass, the half-life of seeds in the soil was associated with CI index (Partial correlation coefficient  = 0.94, *n* = 5, *P*<0.0001), but the half-life of seeds was unrelated to seed mass when CI index was controlled (Partial correlation coefficient  = 0.02, *n* = 5, *P* = 0.98).

**Figure 5:**
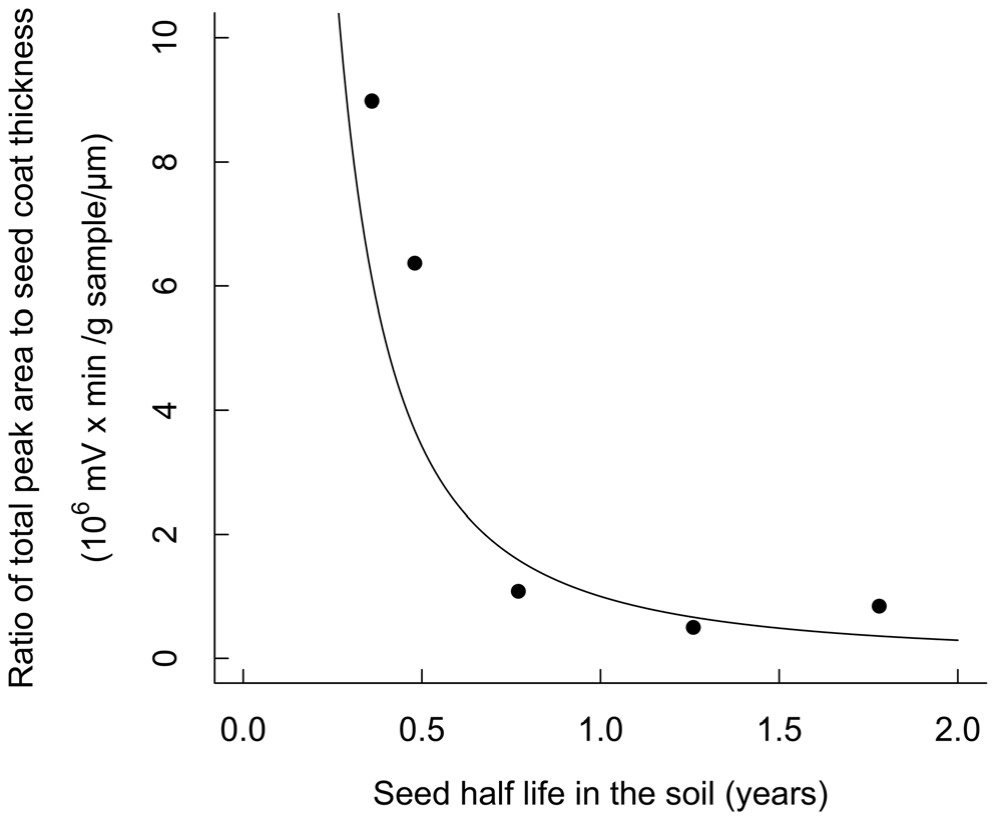
The ratio of seed chemical (total mass-standardized peak area of potential defensive compounds) to physical defence (seed coat thickness) of five *Macaranga* species decreased as seed half-life in the soil increased (log(*Y*) = −1.7732×log(*X*), *n* = 5, *R^2^* = 0.8269, *F _(1,3)_* = 14.33, *P* = 0.032).

Comparing physical and chemical defences, the ratio of total mass-standardized peak area of potential defensive compounds to seed coat thickness (chemical: physical defence) decreased as seed half-life in the soil increased (Figure 5). Linear regression with the PICs of log-seed half-life and log-ratio of total mass-standardized peak area also showed a negative relationship (*Y = −1.78X*, *R^2^ = *0.84, *F _(1, 3)_* = 16.07, *P* = 0.03). Thus, the relative investment in chemical defence to physical defence decreased as seed persistence increased.

## Discussion

This study tested hypotheses for how seed physical and chemical defences, seed mass, light requirement for regeneration and seed persistence are related. We found that variation in defence investment was primarily determined by species habitat requirements. Species that occupy more open environments, and that have longer-lived seed banks invested more in physical defences than more shade-tolerant species. Seed chemical defences, characterized as soluble polar molecules extracted from the seed coat, also varied markedly among species in both their diversity and abundance, and overall, appear to be a more important component of the defensive investment of more shade-tolerant *Macaranga* species.

### Trade-off between seed physical and chemical defence

In general, studies on the allocation of physical and chemical defence in seeds are scarce. A single study, comparing six species of *Lithocarpus* (Fagaceae) with seeds dispersed by scatter-hoarding rodents has reported a trade-off between these defence classes [25]. No direct trade-off was observed in this study (Figure 2); however, the proportional allocation to defensive traits may be contingent on the suite of dispersers and predators that are associated with seeds, and may therefore be more apparent in comparisons among species that have similar capacities to persist in the soil.

The prediction of trade-offs among defensive traits also assumes that resource allocation rules apply at the individual seed level. However, allocation to defence in seeds is a component of the larger investment in seed production [51], [52]. Therefore, both seed size and seed defence investment potentially trade-off with the number of seeds produced.

The efficacy of chemical and physical defences also needs to be evaluated in the context of seed germination. Physical defences protect mature, ungerminated seeds; however, seed coats must soften and crack during seed germination, and seeds may then rely on existing chemical defences or on inducible mechanisms that transform stored precursors to defensive compounds [53], [54]. Future studies are needed to better understand temporal changes in physical and chemical attributes of defence after dispersal and the interactive effect of pathogens and environmental stresses.

### Light requirement for regeneration, seed mass and seed defence

There was large variation in the investment in seed defences among species, which correlated with seed and plant regeneration traits. For seed physical defence, light requirement for regeneration rather than seed mass was the principle determinant of defence traits. Therefore, the results did not support the hypothesis that larger seeds are more reliant on physical defences. In contrast, the results supported the hypothesis that exposure to natural enemies in the soil determines investment in physical defence among species. Seeds of species that occupy more shaded habitats had lower levels of physical protection. Among the study species, shade-tolerant *Macaranga* species in this dataset showed no evidence of delayed germination and therefore may not require the physical defences that permit prolonged persistence in the soil after seed dispersal.

Variation in seed coat thickness may also be related to seed physiology. The thin seed coat of shade-tolerant species may also be explained in part by low desiccation risk in shade habitats. While seed coats play a role in defence, they also serve as a boundary to protect the embryo against fluctuations in humidity and temperature [19]. Shade habitats are moister than large gaps and open sites in tropical forests [55]. Therefore, seeds that germinate in drier high light environments may exhibit thicker seed enclosing structures to regulate water uptake [19], [56] and to prevent rapid desiccation [57]. In contrast, selection for thick seed covers may be low, especially in ever-wet forests, where seeds are not usually exposed to prolonged dry periods.

### Seed chemical defence

The diversity of potential defensive compounds ranged widely, with 1–20 compounds present in the seed coat of the 10 *Macaranga* species. Only one unidentified compound with molecular weight of 330.1 was common to all species. Species grouped according to their similarity in chemical defences did not show associations with either light requirement for regeneration or seed mass. Other factors, such as soil specialization and phylogenetic relatedness among *Macaranga* species, may be correlated to the diversity, quantity and types of potential defensive compounds present in seed coat.

### Seed persistence and seed defence

The results of this study supported the hypothesis that species with seeds that persist longer in the soil rely primarily on seed physical rather than chemical defence. These results were also in agreement with a previous study of six temperate weed species, where Davis et al. (2008) [39] explored the relationship between seed half-life and the relative importance of seed chemical defences, represented by the concentration of *ortho*-dihydroxyphenols, to physical defence, represented by seed coat thickness. They found that relative investment in phenolic defences declined with increasing half-life in the soil. Similarity in tropical tree species and temperate herbaceous species suggest that a reliance on physical defence for long persistence may be universal across different plant groups.

In this study, longer persistent *Macaranga* species had a lower ratio of total abundance of potential defensive compounds (total mass-standardized peak area) relative to seed coat thickness. When the number of potential defensive compounds among species and seed persistence in the soil were considered, the species with longer persistence also produced fewer potential defensive compounds. For the longer persistent species, it might be more resource efficient to produce relatively few potential defensive compounds with high defensive capacity. Alternatively, the few potential defensive compounds produced in species with high persistence may play little role in plant defence and species may rely on ongoing co-evolution between seeds and specialist microbes in the soil as a microbial defence [58].

### Exceptional structure and seed defence of *Macaranga winkleri*

The external surface of enclosing structures of seeds of *Macaranga* species was smooth or only shallowly pitted with one exception, *M. winkleri*. Seed coats of *M. winkleri* had a verrucose surface (Figure 1D). Although many other taxa have similarly sculpted surfaces, the ecological significance of seed coat ornamentation is not well understood. There are, however, two suggested hypotheses that relate to abiotic interactions [59]. First, rough seed coverings could be advantageous in anchoring the seed to the soil (Werker 1997 referred to by [60]). Second, surface sculpturing may control temperature under conditions of high insolation [59]. Since seeds cannot regulate temperature through transpiration, roughness may increase seed surface area, which increases energy exchange with surrounding cooler air.

In addition to abiotic interactions, sculpturing of the seed surface may also play a role in seed microbial defence. The verrucose surface appears not to contribute to seed physical strength, given that *M. winkleri* had the lowest seed fracture resistance. However, the extra surface area may facilitate colonization of beneficial microbes around seeds that may prevent pathogen infection [58], [61]. Among the *Macaranga* species in this study, *M. winkleri* produced the smallest light-dependent seeds [62]. *M. winkleri* seeds were the least physically and chemically protected with low seed strength and only one soluble compound present. However, *M. winkleri* seeds had a half-life of 1.26 years and are abundant in the soil seed bank (Pimonrat Tiansawat, unpublished data). The persistence of *M. winkleri* and evidence of its poor physical and chemical protection may suggest other means of seed defence. Clearly, more work is needed to understand the ecological significance of seed surface characteristics that may influence species regeneration.

In conclusion, for the 10 *Macaranga* species studied here, the lack of correlation between physical defence and chemical defence indicated the absence of a direct trade-off between defence classes. Nevertheless, variation in physical and chemical defence relating to species traits was found among species. Light requirement for regeneration was unrelated to the quantity of potential defensive compounds present in the seed coat. However, light requirement for regeneration was positively correlated with seed coat thickness, when excluding the outlier *M. winkleri*, indicating thinner seed coats in more shade tolerant species. Short exposure to natural enemies after dispersal and physiological benefits may select for thinner seed coats in shade-tolerant species. Finally, this study indicates that seeds with prolonged persistence in the soil relied primarily on seed physical rather than chemical defence.

## Supporting Information

Figure S1 The linear regression through the origin of seed coat thickness (µm) and seed fracture resistance (Newton: N). Seed coat thickness and seed fracture resistance were strongly positively related (*Y = 0.1717X*, *n* = 10, *R^2^* = 0.9667, *F _(1, 9)_* = 206.9, *P*<0.001).
(TIFF)

Figure S2 The linear regression through the origin of the number and total mass-standardized peak area of potential defensive compounds. The number of potential defensive compounds was positively related with total mass-standardized peak area of potential defensive compounds (*Y = 0.893X*, *n* = 10, *R^2^* = 0.9598, *F _(1, 9)_* = 214.7, *P*<0.001). The result corrected for phylogeny was qualitatively similar (*Y = 0.986X*, *n* = 9, *R^2^* = 0.71, *F _(1,8)_* = 19.62, *P* = 0.003).
(TIFF)

Figure S3 Exponential decay function fitted to percent seed germination after burial over time of five *Macaranga* species. For species with two maternal sources in the study, species' seed half-life is the average from the two maternal trees.
(TIFF)

Appendix S1 Fracture resistance measurement and the correlation to seed coat thickness.
(DOCX)

Appendix S2 Extraction of soluble seed phenolic compounds.
(DOCX)

Appendix S3 Analytical method in high performance liquid chromatography (HPLC).
(DOCX)

Appendix S4 Phylogenetic analyses and results of phylogenetic analyses. Table S1, Pearson partial correlation matrix of seed physical defence (seed coat thickness), chemical defence (total mass-standardized peak area), crown illumination index (CI index), and seed mass of nine *Macaranga* species. An outlier, *M. winkleri*, was excluded. The correlation coefficients were not statistically significant at *P*<0.05.
(DOCX)
