# Correction: Investment in Seed Physical Defence Is Associated with Species' Light Requirement for Regeneration and Seed Persistence: Evidence from *Macaranga* Species in Borneo

**DOI:** 10.1371/journal.pone.0144756

**Published:** 2016-01-19

**Authors:** Pimonrat Tiansawat, Adam S. Davis, Mark A. Berhow, Paul-Camilo Zalamea, James W. Dalling

The affiliation for the first author is incomplete, and the affiliations for the second and third authors are incorrect. Please see the complete, correct affiliations here.

Pimonrat Tiansawat is also affiliated with: Department of Biology, Chiang Mai University, Chiang Mai, Thailand.

Adam S. Davis is affiliated with Global Change and Photosynthesis Research Unit, United States Department of Agriculture–Agricultural Research Service, Urbana, Illinois, USA.

Mark A. Berhow is affiliated with the National Center of Agricultural Utilization Research, United States Department of Agriculture–Agricultural Research Service, Peoria, Illinois, USA.
