# Supplementary figures and images for: Investment in Seed Physical Defence Is Associated with Species' Light Requirement for Regeneration and Seed Persistence: Evidence from Macaranga Species in Borneo
Source: PLoS One. 2014 Jun 13;9(6):e99691. doi: 10.1371/journal.pone.0099691 (PMC4057182; doi:10.1371/journal.pone.0099691)

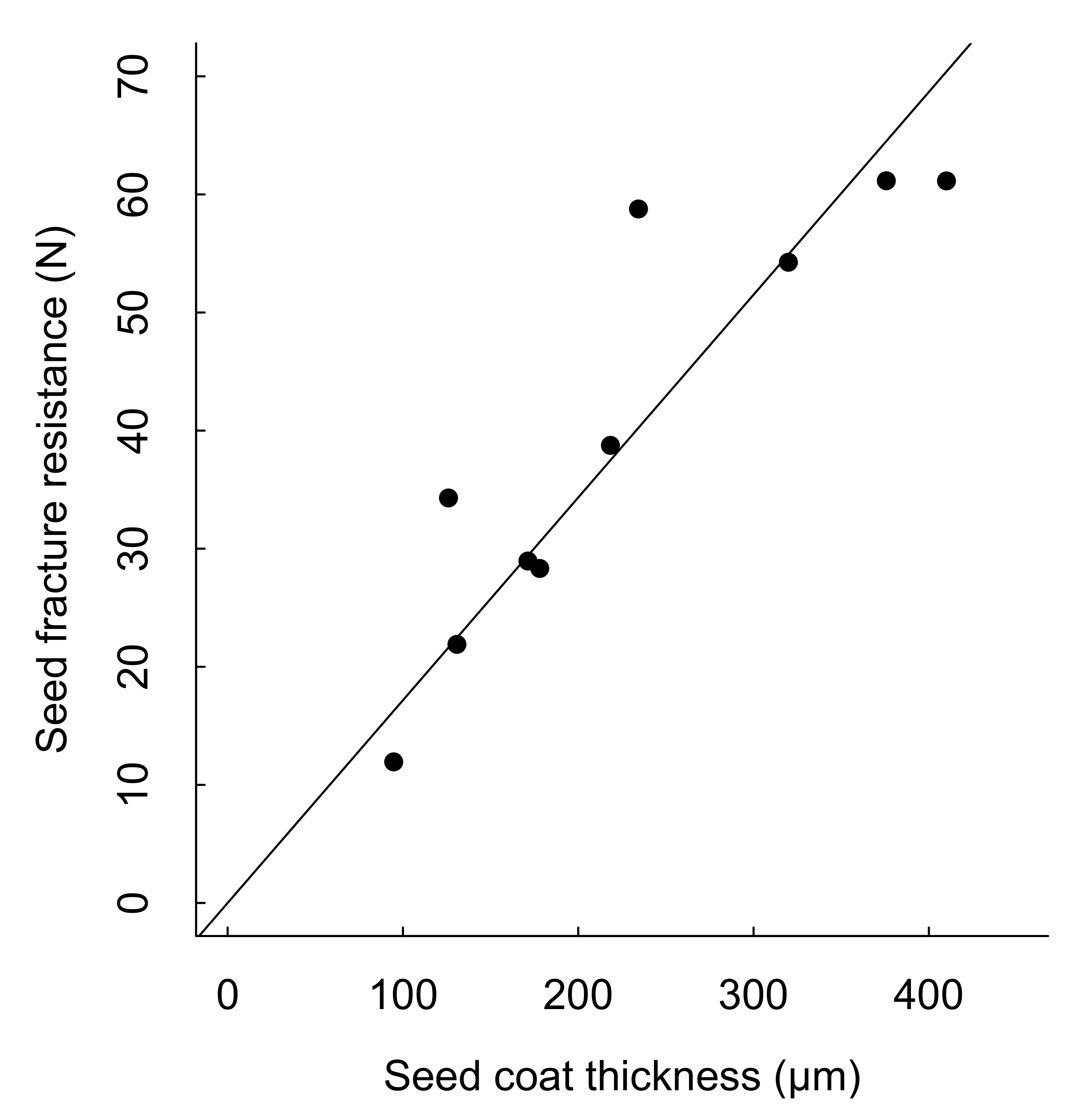

Supplement: Figure S1 — The linear regression through the origin of seed coat thickness (µm) and seed fracture resistance (Newton: N). Seed coat thickness and seed fracture resistance were strongly positively related (Y = 0.1717X, n = 10, R2 = 0.9667, F (1, 9) = 206.9, P<0.001). (TIFF) [file pone.0099691.s001.tiff]

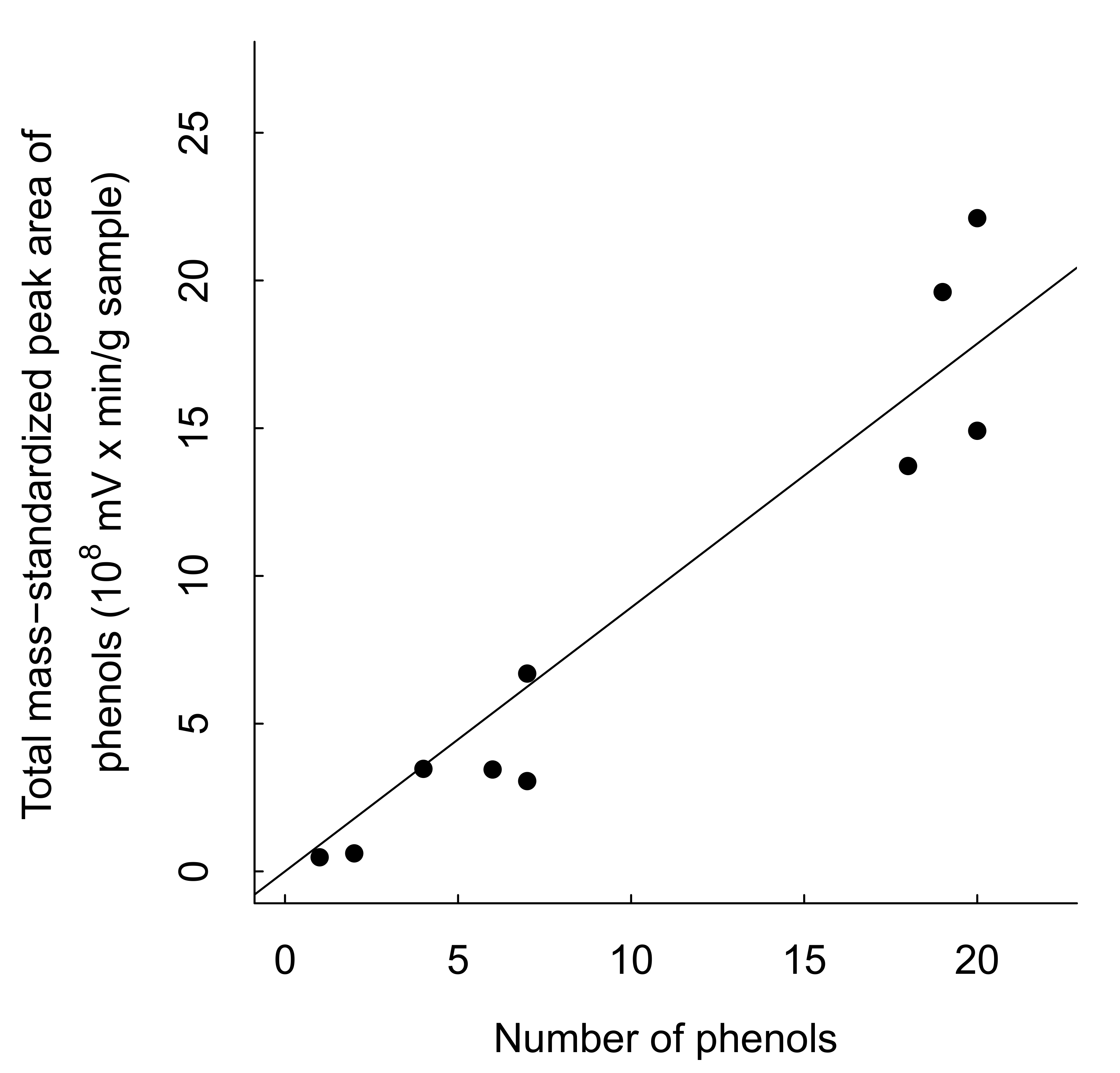

Supplement: Figure S2 — The linear regression through the origin of the number and total mass-standardized peak area of potential defensive compounds. The number of potential defensive compounds was positively related with total mass-standardized peak area of potential defensive compounds (Y = 0.893X, n = 10, R2 = 0.9598, F (1, 9) = 214.7, P<0.001). The result corrected for phylogeny was qualitatively similar (Y = 0.986X, n = 9, R2 = 0.71, F (1,8) = 19.62, P = 0.003). (TIFF) [file pone.0099691.s002.tiff]

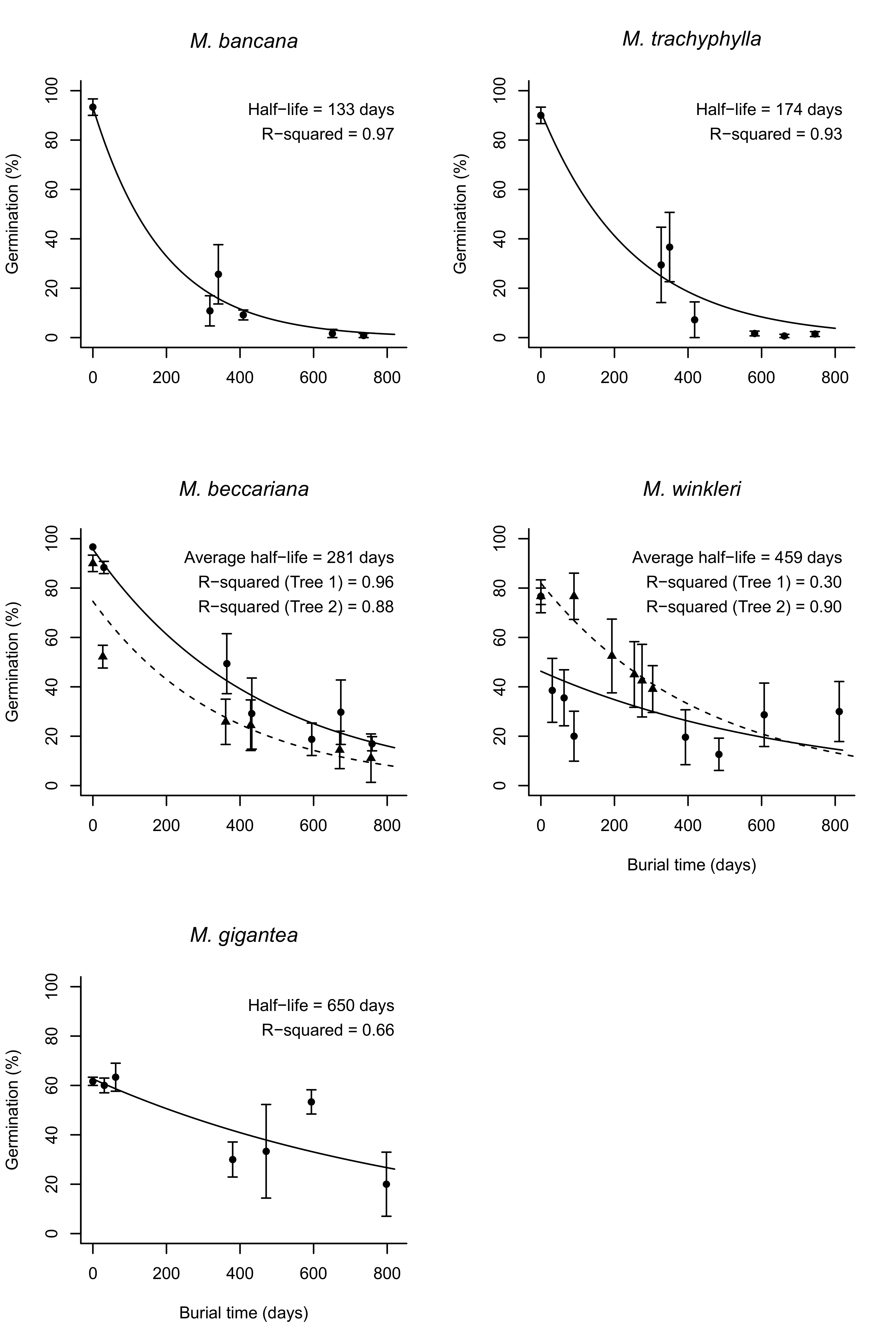

Supplement: Figure S3 — Exponential decay function fitted to percent seed germination after burial over time of five Macaranga species. For species with two maternal sources in the study, species' seed half-life is the average from the two maternal trees. (TIFF) [file pone.0099691.s003.tiff]
