# Supplementary material for: Investment in Seed Physical Defence Is Associated with Species' Light Requirement for Regeneration and Seed Persistence: Evidence from Macaranga Species in Borneo
Source: PLoS One. 2014 Jun 13;9(6):e99691. doi: 10.1371/journal.pone.0099691 (PMC4057182; doi:10.1371/journal.pone.0099691)
Supplement: Appendix S1 — Fracture resistance measurement and the correlation to seed coat thickness. (DOCX) [file pone.0099691.s004.docx]

**Appendix S1**

**Fracture resistance measurement and the correlation to seed coat thickness**

For fracture resistance measurements, seeds were visually inspected and seeds with visible cracks were discarded. Seed fracture resistance was measured as the minimum force required to initiate seed rupture, using an Instron Single Column Testing System Model 3342 (Instron Company, USA). Each seed was loaded between the anvil and the compression probe of the machine and was compressed until the seed coat ruptured. The seed coat rupture created a sudden drop in force, and therefore the force causing the first fracture was recorded. According to seed availability, 10 to 30 seeds of each species were tested, and the mean of fracture resistance was obtained.

The interspecific relationship between seed fracture resistance and seed coat thickness, and between phylogenetically independent contrasts of both traits were analyzed using a simple linear regression. Seed fracture resistance had a strong positive linear relationship with seed coat thickness (Fig. S1). After accounting for phylogeny, contrasts of seed fracture resistance and seed coat thickness were still significantly positively related (*Y= 0.168X, n =* 9*, R^2^ =* 0.59*, F =* 11.58*, P =* 0.009).
