# Supplementary material for: Investment in Seed Physical Defence Is Associated with Species' Light Requirement for Regeneration and Seed Persistence: Evidence from Macaranga Species in Borneo
Source: PLoS One. 2014 Jun 13;9(6):e99691. doi: 10.1371/journal.pone.0099691 (PMC4057182; doi:10.1371/journal.pone.0099691)
Supplement: Appendix S2 — Extraction of soluble seed phenolic compounds. (DOCX) [file pone.0099691.s005.docx]

**Appendix S2**

**Extraction of soluble seed phenolic compounds**

The extraction consisted of two main steps – non-polar chemical removal (de-fatting) in hexane and soluble phenolic compound extraction. In the first step, 5 mL of hexane was added to 0.5 g of ground seed material. The mixture was sonicated for 60 min and left overnight. Before hexane removal on the next day, the mixture was re-sonicated for 60 min. After fat removal, the de-fatted seed samples were divided in three 0.1 g replicates.

In the second step, soluble phenolic compounds were extracted from each replicate in 1.5 ml of methanol overnight. The mixture was sonicated for 60 min after adding methanol and before collecting the methanol supernatant on the next day. To collect the supernatant into an HPLC vial, the supernatant was filtered through a hypodermic syringe filter (0.45 um pore, nylon 66 matrix).
