# Supplementary material for: Investment in Seed Physical Defence Is Associated with Species' Light Requirement for Regeneration and Seed Persistence: Evidence from Macaranga Species in Borneo
Source: PLoS One. 2014 Jun 13;9(6):e99691. doi: 10.1371/journal.pone.0099691 (PMC4057182; doi:10.1371/journal.pone.0099691)
Supplement: Appendix S3 — Analytical method in high performance liquid chromatography (HPLC). (DOCX) [file pone.0099691.s006.docx]

**Appendix S3**

### Analytical method in high performance liquid chromatography (HPLC)

The analysis of soluble phenolic compounds was conducted on a Shimadzu LC-10A HPLC system (two LC-10AT pumps, SIL-10A autosampler, a CTO-10A column oven, a SPD 10AVi variable wavelength UV-Vis detector and a SCL-10Avp system controller, running under Shimadzu LCSolutions version 1.25 chromatography software, Columbia, MD, USA). The column used was an Inertsil ODS-3 reverse phase C-18 column (5 µM, 250 x 4.6 mm, with a Varian metaguard column). For phenolic compound analysis, the initial conditions were 20% methanol and 80% water with 0.05 M phosphoric acid, at a flow rate of 1 ml per minute. The effluent was monitored at 280 and 340 nm on the VWD. After injection (25 µL), the column was held at the initial conditions for two minutes, then developed to 100% methanol in a linear gradient over 55 minutes. Peak detection was at 280 nm. Standards of a variety of phenolics were run at 15 uLs per injection of a 1 mg/mL solution.
