# Supplementary material for: Investment in Seed Physical Defence Is Associated with Species' Light Requirement for Regeneration and Seed Persistence: Evidence from Macaranga Species in Borneo
Source: PLoS One. 2014 Jun 13;9(6):e99691. doi: 10.1371/journal.pone.0099691 (PMC4057182; doi:10.1371/journal.pone.0099691)
Supplement: Appendix S4 — Phylogenetic analyses and results of phylogenetic analyses. Table S1, Pearson partial correlation matrix of seed physical defence (seed coat thickness), chemical defence (total mass-standardized peak area), crown illumination index (CI index), and seed mass of nine Macaranga species. An outlier, M. winkleri, was excluded. The correlation coefficients were not statistically significant at P<0.05. (DOCX) [file pone.0099691.s007.docx]

**Appendix S4**

**Phylogenetic analyses and results of phylogenetic analyses**

To take into account the effects of shared evolutionary history, phylogenetic analyses were also conducted on trait comparisons. The topology of the phylogenetic tree was generated using the web interface of Phylomatic version 3 [1] with a megatree based on Smith et al. (2011) [2]. All one-daughter nodes in the phylogenetic tree were removed. There is no branch length information for *Macaranga*; therefore, all branch lengths were initially converted to a branch length of 1 (non-ultrametric tree). To convert the non-ultrametric tree to an ultrametric tree, representing all extant taxa, the branch lengths were estimated using a semi-parametric method based on penalized likelihood using package *ape* version 3.0-8 (*choronopl*; [3]) with an arbitrary smoothing parameter of 1, which controls the trade-off between smoothness, and goodness-of-ﬁt of the data to the saturated model (different divergence times of all branches) and to the clock model (no variation in the divergence rate) [4]. The length of the tree was adjusted by the height of the non-ultrametric tree (height = 7). Then, values of phylogenetically independent contrasts (PICs) of each trait were obtained using package *ape*. The contrasts of each trait were checked to ensure they met the assumption of standardization by branch lengths using Pearson correlation tests. If the assumption is met, then there is no correlation between the PICs and their standard deviations [5]. The PICs between traits of interest were analysed using Pearson’s correlation, partial correlation and linear regression through the origin [5].

**Results**

Overall, the results of qualitatively similar to cross-species analyses. There was no linear relationship between PICs of seed coat thickness and PICs of total peak area of potential defensive compounds (Figure 2). The CI index was significantly negatively correlated with seed mass using a regression analysis of PICs (*Y = -19.26X*, *n* = 9, *R^2^* = 0.47, *F* = 8.86, *P* = 0.02). Because the strong correlation between seed mass and CI index, their association with thickness of seed coat was examined using partial correlation. When controlling for CI index, seed coat thickness was uncorrelated with seed mass (Table S1). When controlling for seed mass, and excluding the outlier *M. winkleri*, seed coat thickness was positively associated with CI index (Table S1). The relationship between seed coat thickness and CI index was positive, but was no significant at *P* < 0.05 (Table S1). The total mass-standardized peak area was not correlated with either CI index or seed mass (Table S1).

**References**

1. Webb CO, Donoghue MJ (2005) Phylomatic: Tree assembly for applied phylogenetics. Mol Ecol Notes 5: 181-183.

2. Smith SA, Beaulieu JM, Stamatakis A, Donoghue MJ (2011) Understanding angiosperm diversification using small and large phylogenetic trees. Am J Bot 98: 404-414.

3. Paradis E, Claude J, Strimmer K (2004) APE: analyses of phylogenetics and evolution in R language. Bioinformatics 20: 289-290.

4. Sanderson MJ (2002) Estimating absolute rates of molecular evolution and divergence times: A penalized likelihood approach. Mol Biol Evol 19: 101-109.

5. Garland T, Harvey PH, Ives AR (1992) Procedures for the analysis of comparative data using phylogenetically independent contrasts. Syst Biol 41: 18-32.

**Table S1** Pearson partial correlation matrix of seed physical defence (seed coat thickness), chemical defence (total mass-standardized peak area), crown illumination index (CI index), and seed mass of nine *Macaranga* species. An outlier, *M. winkleri*, was excluded. The correlation coefficients were not statistically significant at *P* < 0.05.

|  | Phylogenetic analysis | |
| --- | --- | --- |
|  | CI index | Seed mass |
| Seed coat thickness | 0.48 | -0.19 |
| Total mass-standardized peak area | 0.03 | -0.31 |
